# Continuous administration of corticosteroids in septic shock can reduce risk of hypernatremia

**DOI:** 10.1186/cc13429

**Published:** 2014-03-17

**Authors:** L Mirea, R Ungureanu, D Pavelescu, IC Grintescu, C Dumitrache, I Grintescu, D Mirea

**Affiliations:** 1 Clinical Emergency Hospital of Bucharest, Romania; 2 CI Parhon National Institute of Endocrinology, Bucharest, Romania; 3 Elias University Emergency Hospital, Bucharest, Romania

## Introduction

Although the administration of hydrocortisone in septic shock generates adverse effects, the risk of corticosteroid-induced hypernatremia may be reduced by continuous administration of the drug [1][2].

## Methods

A total of 171 patients with septic shock were randomized into three study groups: group A (*n *= 58), 200 mg/day hydrocortisone hemisuccinate in four doses; group B (*n *= 59), same dose of hydrocortisone hemisuccinate in continuous administration; group C (*n *= 54), no hydrocortisone hemisuccinate. Mean serum sodium values, the number of hypernatremia episodes and variations in serum sodium (Na var) were investigated for 7 days. The local ethics committee approved the study.

## Results

There were no differences between the three groups at the beginning of the study regarding demographic data and the clinical characteristics. Mean values of natremia were normal in group C (140.35 ± 7.390 mEq/l to 144.79 ± 8.338 mEq/l) during the study period. High mean values appeared on day 4 in group A (147.21 ± 8.470 mEq/l to 149.37 ± 8.973 mEq/l on day 7) and on day 5 in group B (146.36 ± 8.272 mEq/l to 147.70 ± 8.865 mEq/l). Na var was 8.59 ± 5.960 mEq/l (-8 and 21 mEq/l) in group A, 6.63 ± 7.609 mEq/l (-17 and 23 mEq/l) in group B and 4.54 ± 7.455 mEq/l (-12 and 22 mEq/l) in group C. This variation is statistically significant when groups A and B are compared with group C (*P *= 0.012) and when only group A is compared with group C (*P *= 0.0019). The risk of hypernatremia after hydrocortisone hemisuccinate was almost three times higher than that of patients who did not receive this drug (RR 2.82, 1.35 <OR <5.90, *P *= 0.0041) and slightly higher when HHS was delivered as a bolus (RR 3.08, 1.32 <OR <7.25, *P *= 0.0071).

## Conclusion

Continuous administration of hydrocortisone hemi- succinate in septic shock is associated with a lower risk of hypernatremia than bolus administration.
